# Durability of Adaptive Immunity in Immunocompetent and Immunocompromised Patients Across Different Respiratory Viruses: RSV, Influenza, and SARS-CoV-2

**DOI:** 10.3390/vaccines12121444

**Published:** 2024-12-22

**Authors:** Achilleas Livieratos, Lars Erik Schiro, Charalambos Gogos, Karolina Akinosoglou

**Affiliations:** 1 Independent Researcher, 15238 Athens, Greece; 2 Independent Researcher, 0284 Oslo, Norway; larserik.schiro@outlook.com; 3 Department of Medicine, University of Patras, 26504 Rio, Greece; cgogos@med.upatras.gr (C.G.); akin@upatras.gr (K.A.); 4 Department of Internal Medicine and Infectious Diseases, University General Hospital of Patras, 26504 Rio, Greece

**Keywords:** immunization, immunocompromised, RSV, SARS-CoV-2, influenza, adaptive immunity

## Abstract

Background/Objectives. Research on respiratory virus immunity duration post-vaccination reveals variable outcomes. This study performed a literature review to assess the efficacy and longevity of immune protection post-vaccination against SARS-CoV-2, influenza, and respiratory syncytial virus (RSV), with a focus on immunocompromised populations. Specific objectives included examining humoral and cellular immune responses and exploring the impact of booster doses and hybrid immunity on extending protection. Methods. A literature review was conducted focusing on studies published from January 2014 to November 2024. The search targeted adaptive immunity post-vaccination, natural immunity, and hybrid immunity for SARS-CoV-2, influenza, and RSV. Selection criteria emphasized human populations, adaptive immunity outcomes, and immunocompromised individuals. The PICO framework guided the analysis, culminating in a detailed review of 30 studies. Results. SARS-CoV-2 vaccines exhibited robust initial antibody responses, which waned significantly within six months, necessitating frequent boosters. Influenza and RSV vaccines similarly showed declines in immunity, though some influenza vaccines demonstrated moderate durability. Hybrid immunity, arising from combined natural infection and vaccination, provided more resilient and lasting protection than vaccination alone, especially against emerging variants. Immunocompromised individuals consistently exhibited reduced durability in adaptive immune responses across all studied viruses. Challenges include rapid viral mutations, limiting the broad protection of current vaccines. Conclusions. Immune durability varies significantly across virus types and patient populations. Frequent boosters and hybrid immunity are critical to optimizing protection, particularly for vulnerable groups. The findings underscore the need for adaptable vaccination strategies and advancements in vaccine design to counter rapidly mutating respiratory pathogens effectively.

## 1. Introduction

The rise of a “tridemic” season, characterized by the concurrent circulation of the respiratory viruses SARS-CoV-2, influenza, and RSV, highlights the importance of identifying immune responses to combat severe disease outcomes [1,2]. These infections frequently lead to prolonged hospitalizations, respiratory failure, and poor clinical responses to initial antibiotic treatments, particularly in vulnerable populations, including immunocompromised individuals [3,4]. During the 2024–2025 respiratory season, the United States (U.S.) has reported approximately 1.5 million COVID-19 cases, compared to 1.2 million in the European Union (EU), with hospitalization rates in both regions highest among individuals aged 65 and older [1,2]. Influenza cases are slightly higher in the U.S., at an estimated 2.3 million, versus 1.8 million in the EU, with both regions experiencing similar hospitalization trends [1,2]. In the U.S., RSV has caused around 800,000 cases, while the EU has reported over 700,000 cases; children under 4 years old are the most affected group in both regions, contributing to 40% of RSV-related hospitalizations in the EU [1,2]. Mortality for all three viruses—COVID-19, influenza, and RSV—remains concentrated among older adults, and while precise fatality counts are unavailable, cumulative respiratory virus mortality in the EU is within typical seasonal levels, mirroring trends in the U.S. [1,2]. The overlapping peaks of these viral outbreaks place a significant burden on healthcare systems and necessitate precise vaccination and treatment strategies [3,4]. Knowledge of adaptive immunity durability, particularly in this context, is pivotal in reducing severe outcomes and guiding evidence-based decisions on vaccination schedules, booster doses, and tailored interventions for at-risk populations [5].

Early data indicate that mRNA-based SARS-CoV-2 vaccines elicit high levels of neutralizing antibodies [6,7,8,9]. This is accompanied by proportional cellular responses following a booster dose and is associated with marked declines in antibody levels within six months for immunocompetent individuals [6,7,8,9]. These data indicate a potential reduction in the protective effect in the long term following immunization, which, when coupled with the impaired immune system of immunocompromised patients, could be detrimental to clinical outcomes [10,11,12]. This waning immunity is especially concerning in populations like healthcare workers and dialysis patients, who experience rapid antibody declines, correlating with an increased risk of breakthrough infections [13,14]. As a result, further booster doses have been recommended to restore immunity and reduce the risk of infection [15,16,17,18,19,20,21].

Interestingly, hybrid immunity, which is the result of exposure to both natural infection and vaccination, is reported to promote more durable protection compared to immunization alone [22,23,24,25,26]. Patients with prior SARS-CoV-2 infections exhibit higher levels of neutralizing antibodies post-vaccination, highlighting the potential benefits of hybrid immunity in providing longer-lasting protection, though with the risk of severe illness from the natural infection itself [22,23,24,25,26].

In addition to the widely investigated SARS-CoV-2 vaccines, research on the recently developed RSV vaccines has demonstrated robust neutralizing antibody responses, particularly in older adults who are at increased risk of severe RSV infections [27,28,29,30]. However, as with SARS-CoV-2 vaccines, antibody levels have shown a tendency to wane within six months, potentially even faster in immunocompromised individuals, prompting concerns about a potential need for booster doses to sustain long-term immunity against RSV as well [31]. This decline mirrors that seen with other viral vaccines, further suggesting that periodic boosters may be necessary to maintain adequate protection [6,7,8,9,13,14,15,16,31]. Preliminary data on RSV vaccines suggest that similar mechanisms of cellular immunity may be at play, with robust cellular responses helping reduce disease severity despite lower antibody titers [32,33,34,35].

Lastly, it is widely recognized that influenza vaccination remains the cornerstone of preventive strategies against seasonal and pandemic flu. Studies suggest that immunity can wane significantly within six months post-vaccination, especially in older adults, where immune senescence may play a role [36,37,38]. The decay of both neutralizing antibodies and T-cell memory responses has been documented, calling into question the duration of protection afforded by current vaccine formulations [39]. Several studies comparing inactivated and live vaccines have demonstrated that adaptive immunity, such as T-cell memory, crucial for long-term immunity, may persist longer in recipients of live vaccines than in those receiving inactivated ones [40,41,42]. However, immune escape through antigenic drift and wrong target antigen may limit the effectiveness of both types of vaccines, necessitating annual reformulation and revaccination [39,43,44,45]. Recent advancements, such as cell-based vaccines, offer promising avenues for enhanced durability. These vaccines utilize cell culture technologies to improve the stability of the antigen and reduce the reliance on egg-based production, which can introduce variability in immune responses [46,47,48]. Nevertheless, long-term studies are still needed to fully assess how these vaccines perform in terms of durable immunity.

In immunocompetent individuals, adaptive immunity following vaccination against SARS-CoV-2, influenza, and RSV persists for approximately 6–12 months, 12–18 months, and 6 months, respectively, suggesting that vaccination elicits an immune response comparable to that achieved through natural infection [49,50,51,52,53,54,55,56,57,58]. Notably, in this population, hybrid immunity appears to confer a shorter duration of adaptive immunity against SARS-CoV-2 and influenza, while data on the duration of hybrid immunity against RSV remains unavailable [59,60,61,62,63]. In contrast, immunocompromised individuals exhibit a diminished adaptive immune response to vaccination against SARS-CoV-2 and influenza compared to their immunocompetent counterparts, with no current data addressing the efficacy of RSV vaccination in this population [64,65,66,67,68]. Upon natural infection, immunocompromised individuals generate a short-term adaptive immune response to SARS-CoV-2, influenza, and RSV, though this response is consistently attenuated relative to immunocompetent individuals [69,70,71,72,73]. Hybrid immunity in immunocompromised individuals, however, appears to induce a more robust adaptive immune response against both SARS-CoV-2 and influenza compared to either vaccination or natural infection alone [74,75,76,77,78]. Despite this advantage, the inherent risks associated with natural infection in immunocompromised populations render this immunization strategy impractical in most clinical scenarios.

In summary, the durability of adaptive immunity in vaccinated patients plays a critical role in shaping effective long-term strategies against respiratory pathogens such as SARS-CoV-2, RSV, and influenza. As immune responses diminish over time, particularly in the face of rapidly evolving viral strains, it becomes crucial to assess the impact of booster doses and the mechanisms underlying immune memory. We will, therefore, provide a comparative analysis of the current evidence on immune persistence across these three major viral targets, exploring how vaccination strategies can be optimized to ensure sustained protection, particularly for immunocompromised patients.

## 2. Materials and Methods

We conducted an extensive literature search using PubMed, Embase, and Scopus of manuscripts published in the last decade (January 2014–November 2024). The search strategy employed the query terms RSV OR Influenza A OR Influenza B OR SARS-CoV-2 AND Adaptive Immunity AND Vaccination OR Natural Immunity OR Hybrid Immunity AND Immunocompromised. This research was structured using the PICO framework, which includes population, intervention, comparison, and outcome. The population includes immunocompromised individuals, such as those with cancer, undergoing organ transplantation, or living with autoimmune diseases. The intervention focuses on vaccination or natural immunity, including hybrid immunity, while the comparison involves immunocompetent populations. The primary outcome focused on the efficacy of the adaptive immune response, such as the durability of immunogenicity outcomes.

An initial search yielded 1322 potentially relevant articles. Studies were included if they focused on human populations, addressed adaptive immunity concerning RSV, Influenza A, Influenza B, or SARS-CoV-2, and examined vaccination, natural immunity, or hybrid immunity in immunocompromised and immunocompetent individuals. Additional criteria for inclusion required that the studies be published in peer-reviewed journals and written in English. Exclusions were applied to articles focusing on animal studies, research irrelevant to adaptive immunity or the specified diseases, articles not available in full text, or published in languages other than English. Duplicate publications and incomplete conference abstracts were also excluded.

**Figure 1:**
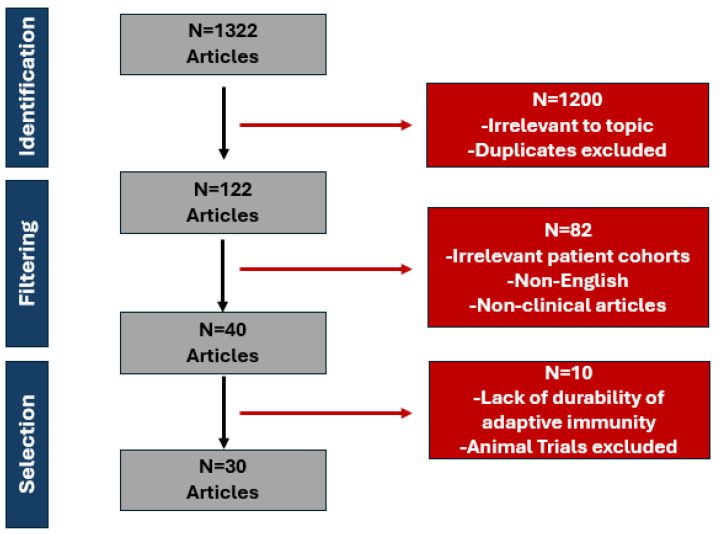
Selection process of RSV, Influenza A/B, and SARS-CoV-2 articles.

Out of these, 1200 articles were excluded for being off-topic or involving animal studies. For example, excluded studies focused solely on epidemiology, virology, or unrelated aspects of disease without linking to the immune response or vaccination in humans. Other articles excluded prioritized immunity frameworks or mechanisms not included in the study’s specific focus (e.g., innate immunity without reference to adaptive immunity). A detailed analysis of the final 122 articles resulted in the elimination of another 92 studies due to their focus on patient cohorts that were not relevant to our research. (see Figure 1). Three investigators separately reviewed and manually examined the literature, resolving any disagreements through collaboration.

## 3. Results

A total of 30 articles were finally evaluated. Among those, the durability of adaptive immunity variation was significant between different articles. A total of 15 articles were included in the immunocompetent analysis in Table 1, examining vaccine-induced, natural, and hybrid immunity across three diseases: SARS-CoV-2, Influenza A/B, and RSV. The sample sizes of the studies ranged from 22 to 5,724,810 participants, with most studies focusing on SARS-CoV-2 (7 studies), followed by Influenza (6 studies) and RSV (2 studies) [49,50,51,52,53,54,55,56,57,58,59,60,61,62,63]. The duration of immune responses varied across studies, with vaccine-induced immunity being assessed for durations ranging from 6 months to 18 months, natural immunity from 3–4 months to >15 months, and hybrid immunity for periods between 2 months and 12 months [49,50,51,52,53,54,55,56,57,58,59,60,61,62,63]. Studies investigating vaccine-induced immunity measured outcomes such as memory B cell responses, neutralizing antibody levels, T cell responses, and time until infection. Studies on natural immunity assessed antibody levels, memory B cells, T cell responses, and hemagglutination-inhibition antibody levels, with varying durations of response. Hybrid immunity, due to immunization and prior infection combined, was mainly evaluated by examining repeat PCR-confirmed infections, antibody levels, and T cell activity. Collectively, these studies focused on capturing diversity regarding the durability of adaptive immunity (cellular and humoral).

A total of 15 studies were included in this immunocompromised analysis in Table 2, focusing on vaccine-induced, natural, and hybrid immunity across SARS-CoV-2, Influenza A/B, and RSV [64,65,66,67,68,69,70,71,72,73,74,75,76,77,78]. The studies primarily investigated immune responses in individuals with compromised immune systems, such as those with hematological malignancies, solid organ transplants, immunosuppressive therapy, and primary immunodeficiencies. Sample sizes varied widely, ranging from as few as 5 participants to as many as 6,391,634. Vaccine-induced immunity was assessed in 5 studies, with immunity durations ranging from <6 months to under 12 months, and findings included rates of confirmed infections and deaths, as well as T cell assays and spike protein antibody levels [64,65,66,67,68]. Natural immunity was examined in 5 studies, with sample sizes ranging from 5 to 196 participants and immunity durations from 1 month to 9 months [69,70,71,72,73]. This immunity was evaluated through the measurement of spike protein antibody levels, T cell activation, and cytokine secretion, with RSV studies also measuring pre-fusion F antibody titers. Hybrid immunity was investigated in 5 studies, with sample sizes ranging from 90 to 488 participants and durability spanning from 28 days to 8 months [74,75,76,77,78]. These studies focused on spike protein antibody levels, T cell activation, and interferon-gamma secretion, with one study also assessing seroprotection and seroconversion rates for influenza. The largest study, conducted by Szekanecz et al., included over 6 million participants and examined rates of confirmed infections and deaths over a 3–6-month period, showing improvements in immunity following booster doses [64]. These findings provide essential conclusions about the immune reactions of vulnerable patients and underscore the relevance of continuing monitoring. Collectively, these two tables offer a comparative perspective on the durability of adaptive immunity across the three different viral infections in immunocompetent and immunocompromised patients.

## 4. Discussion

### 4.1. Vaccine-Induced Immunity

For immunocompetent populations, protection against SARS-CoV-2, influenza, and RSV following immunization generally exhibits a durability of six to twelve months, with SARS-CoV-2 vaccines showing high initial antibody levels that wane within six months (Table 1). Influenza vaccines provide a modestly extended immunity in some studies, reaching up to 18 months, while RSV vaccines demonstrate protection expected to last about six months [49,50,51,52,53]. In immunocompromised populations, the durability of vaccine-induced immunity is notably shorter (Table 2). Studies indicate that SARS-CoV-2 vaccine immunity in these individuals often declines after three to six months, with some benefits observed from booster doses [64,65,66]. Similarly, influenza immunity fades within six months, and RSV vaccines, while promising, have demonstrated only limited data in these populations [67,68]. This trend suggests that while vaccines are effective across both groups, immunocompromised individuals may require more frequent boosters to maintain protective immunity, especially as new variants emerge in pathogens like SARS-CoV-2 and influenza.

Immunocompromised individuals represent a heterogeneous group with varying degrees of immune dysfunction, which influences the way their immune systems respond to infections, vaccines, and immune challenges. The efficacy of vaccines varies among different immunocompromised states, with some groups showing good responses (>60% compared to healthy controls) and others poor (<40%) [79]. Strategies to improve vaccine efficacy include proper timing, booster doses, and newer immunological approaches [79,80]. For patients with poor vaccine responses, additional measures such as high-dose vaccines, revaccination when less immunosuppressed, and long-acting monoclonal antibodies may be considered [79].

Individuals undergoing immunosuppressive therapy or those with solid organ transplants have impaired adaptive immune responses, particularly in the context of T cell and B cell activation, as well as antibody production [64,65,66,67,68]. In such populations, vaccine-induced immunity often shows diminished effectiveness, with reduced antibody responses and T-cell activation compared to healthy individuals [64,65,66,67,68]. This is especially evident in studies of SARS-CoV-2 and influenza, where immunity typically wanes more quickly in immunocompromised individuals [64,65,66,67,68]. Hybrid immunity has been shown to be more effective in some immunocompromised individuals, as observed in studies like Nazaruk et al. and Al-Dury et al. [74,77]. However, even in these cases, the durability of the response can vary significantly depending on the underlying immune dysfunction.

As outlined in Table 1 and Table 2, mRNA vaccines, like those developed for SARS-CoV-2, are known for their rapid production and ability to elicit strong antibody and T-cell responses, though their protection wanes over time, necessitating booster doses [81,82,83]. Viral vector vaccines, such as adenovirus-based platforms, induce both cellular and humoral immunity but may face challenges like pre-existing immunity to the vector [81,82,83]. Inactivated vaccines offer safety and stability, especially for immunocompromised individuals, but often require adjuvants and repeated doses to maintain immunity. Live attenuated vaccines closely mimic natural infections, providing robust protection, but their applicability is not prevalent in patients with weakened immunity [81,82,83]. Overall, these diverse platforms highlight the tailored approaches required to combat respiratory viruses with varying immune evasion strategies and mutation rates [81,82,83].

The development of effective vaccines for these viruses requires a nuanced approach to antigen configuration [81,82]. Previous research highlights the role of local IgA as a crucial component of mucosal immunity, particularly in the upper respiratory tract, where the virus initially infects [81,82]. For instance, nasal IgA has shown effectiveness in preventing reinfection by pathogens like RSV, suggesting that next-generation vaccines could focus on enhancing mucosal IgA responses through targeted delivery methods, such as intranasal vaccines [81,82].

Respiratory viruses like SARS-CoV-2, influenza, and RSV primarily rely on systemic immune responses, with a focus on circulating antibodies and cellular immunity [81,82,83]. However, their immunity often wanes quickly, necessitating frequent boosters, especially as these viruses mutate rapidly. In contrast, enteric viruses such as rotaviruses and Vibrio cholerae engage the mucosal immune system more robustly, particularly through secretory IgA at gut surfaces [84]. This localized response not only provides direct protection against viral adherence and invasion but also establishes long-lasting immune memory, sometimes independent of sustained antibody levels [84]. Additionally, oral vaccines for enteric viruses are specifically designed to stimulate mucosal sites and can confer partial immunity to distant mucosae, a benefit less evident in respiratory virus immunity [84]. Interestingly, the frequent antigenic changes in respiratory viruses and their reliance on systemic responses make achieving durable immunity more challenging compared to the relatively stable and targeted mucosal defense against enteric viruses [81,82,83].

### 4.2. Natural Immunity

Natural immunity in immunocompetent populations offers variable durability depending on the virus, with influenza immunity lasting considerably longer than SARS-CoV-2 or RSV. Some studies show influenza immunity extending beyond 15 months, with a minority observing up to seven years of sustained protection [56,57]. For SARS-CoV-2, natural immunity can last between six to twelve months, while for RSV, antibody responses decline even more rapidly [54,55,58]. Among immunocompromised patients, natural immunity is less reliable, typically showing a significant reduction in durability [69,70,71,72,73]. SARS-CoV-2 natural immunity wanes within six months in immunocompromised groups, with only a few months of efficacy observed in some cases [69,70,71]. Influenza immunity is also shorter-lived, with antibody levels often returning to baseline within a few months post-infection [72]. This variability in natural immunity durability highlights the immune system’s capacity to remember influenza over a longer period than SARS-CoV-2 or RSV, possibly due to repeated exposures to influenza over time, even in immunocompromised individuals. However, for SARS-CoV-2 and RSV, frequent reinfections and waning immunity necessitate additional intervention in vulnerable populations [82,84].

The rapid appearance of different SARS-CoV-2 strains alongside the complex interplay of natural immunity reflects a nuanced landscape of immune responses and clinical outcomes [85,86]. Successive variants such as Delta and Omicron have demonstrated varying pathogenic and immune-evasive characteristics, impacting both the transmissibility and severity of SARS-CoV-2 across populations [85,86]. Delta, for instance, was associated with a higher viral load and severe clinical outcomes, including increased hospitalization and ICU admissions, likely due to its capacity to evade initial immune responses through mutations that enhance infectivity and immune escape mechanisms [85,86]. In contrast, Omicron, despite its high transmissibility, generally led to milder infections, suggesting a shift in viral adaptation toward increased spread but reduced virulence [85,86]. However, Omicron’s evasion of vaccine-induced and natural immunity underlines its ability to reduce the efficacy of pre-existing immunity, especially in individuals without recent exposure or booster doses [85,86].

Natural immunity, while robust after initial SARS-CoV-2 infections, varies considerably in durability and breadth across individuals [85,86]. Studies show that prior infection induces significant antibody and cellular responses, with memory T cell populations providing sustained cross-reactivity against subsequent variants [85,86]. Yet, the extent and longevity of immunity are influenced by factors such as viral load, individual immune system status, and whether immunity is solely natural or hybrid (natural plus vaccine-induced) [85,86]. RSV reinfection with similar strains occurs despite natural infection inducing strong humoral immunity due to effective immunomodulatory mechanisms [87]. Individual differences in innate antiviral immunity exist, with endogenous interferons and TNF-α contributing to resistance against viral infections [88].

### 4.3. Hybrid Immunity

Hybrid immunity—combining vaccine-induced and natural immunity—stands out across both tables as an effective approach, especially for SARS-CoV-2 and influenza. In the general population, hybrid immunity for SARS-CoV-2 maintains about 50% of its initial efficacy six months post-vaccination, while influenza shows durability extending up to one year in some cases [59,60,61,62,63]. Immunocompromised individuals similarly benefit from hybrid immunity, though its durability is often less pronounced [74,75,76,77,78]. For SARS-CoV-2, hybrid immunity can enhance protection beyond vaccination alone, lasting up to eight months in some studies [74]. However, this advantage is less consistent for influenza and under-researched for RSV, though some data suggest hybrid approaches could confer a degree of durable protection.

Studies have shown that hybrid immunity leads to stronger and more durable antibody and T-cell responses compared to immunity generated solely by infection or vaccination [85]. Hybrid immunity’s enhanced protection is attributed to its ability to address variant-induced immune escape more effectively, as it triggers a wider range of immune memory and antibody responses [85]. This breadth makes hybrid immunity especially valuable in managing SARS-CoV-2’s evolving strains, where traditional immunity can be limited in its effectiveness [85]. While RSV and influenza also show some benefits from hybrid exposure, the effect is less pronounced than in SARS-CoV-2 due to factors such as RSV’s immune-evasive mechanisms and influenza’s high mutation rate [85].

### 4.4. Emerging Insights

A comparison of these tables reveals several key insights. First, immunocompromised individuals generally exhibit a more rapid decline in immunity across all types and pathogens, indicating a need for more frequent booster doses and enhanced vaccine protocols (Table 2). Second, SARS-CoV-2 vaccines appear to elicit stronger initial antibody responses, but these responses wane quickly, making frequent boosting crucial, particularly for immunocompromised populations [64,65,66,67,68]. Third, the advantage of hybrid immunity is consistent across both groups, reinforcing its value as a durable protection method, especially in populations vulnerable to severe infections [59,60,61,62,63,74,75,76,77,78]. Finally, while influenza immunity shows greater persistence, the variability in RSV and SARS-CoV-2 responses underscores the evolving challenge of developing long-lasting vaccines for rapidly mutating viruses. These findings suggest that tailored vaccination schedules, possibly integrating natural immunity in a controlled manner, could optimize protection, especially in those with compromised immune systems.

Current vaccines for rapidly replicating mucosal respiratory viruses often fail to elicit complete and durable protective immunity [64,65,66,67,68]. This is partly due to the challenge of mimicking respiratory infection through vaccination, resulting in robust systemic responses but poor mucosal protection [79,80]. Developing effective next-generation vaccines requires consideration of various factors, including antigen configuration, dosage, adjuvants, and vaccination routes [80,81]. Additionally, while strong immune responses are necessary for viral clearance, they must be balanced to prevent lung damage and maintain pulmonary homeostasis [80,81]. This is particularly evident in severe COVID-19 cases, where lung autopsies reveal extensive damage due to immune-mediated pathology [80,81]. Thus, balancing the immune activation to clear infection without triggering harmful inflammation is critical. The evolution and rapid antigenic drift of influenza and SARS-CoV-2 present ongoing challenges. Unlike the relatively stable measles virus, these respiratory pathogens undergo frequent mutations, complicating vaccine design [80,81]. Targeting conserved antigenic regions across strains, such as the influenza virus’s hemagglutinin stem or SARS-CoV-2 spike protein regions that are less prone to mutation, may offer a pathway to broader and more durable immunity [80,81].

RSV infection presents a notable challenge in adaptive immunity, as it typically induces high levels of antibodies, yet immunity remains short-lived [89,90,91]. Reinfections with RSV are common across all age groups, a phenomenon attributed to the limited durability of RSV-specific antibodies and inadequate memory B cell responses [89,90,91]. These characteristics suggest that the adaptive immune response to RSV does not provide sustained protection. RSV evades the immune system through mechanisms that impair key signaling pathways [89,90,91]. This impairment leads to an attenuated immune memory that affects both T and B cell responses [89,90,91].

Immunity against SARS-CoV-2, in contrast, results from an intricate interplay between innate and adaptive immune mechanisms [85,92,93,94]. SARS-CoV-2 infection activates a vigorous innate immune response, with a marked increase in interferon production that initially helps curb viral replication [85,92,93,94]. However, in severe cases, this immune activation can become excessive, leading to a cytokine storm that exacerbates lung injury and contributes to severe clinical outcomes [85,92,93,94]. Unlike RSV, SARS-CoV-2 generally induces lasting adaptive immunity, particularly in individuals who experience severe infection or are vaccinated [85,92,93,94]. High titers of neutralizing IgG antibodies are produced, targeting the spike protein’s receptor-binding domain, which is also the primary focus of SARS-CoV-2 vaccines [85,92,93,94]. Despite the development of a strong adaptive response, SARS-CoV-2 can mutate rapidly, as evidenced by the emergence of variants like Delta and Omicron [85,92,93,94]. These mutations can reduce vaccine efficacy by partially evading neutralizing antibodies, resulting in breakthrough infections among vaccinated individuals [85,92,93,94]. Nonetheless, T-cell responses tend to remain robust, which reduces the severity of reinfections and contributes to overall protection against severe disease.

The influenza virus, however, operates under a distinct immunological paradigm. Natural infection with influenza virus elicits a robust strain-specific immune response characterized by the production of both IgG and IgA antibodies [86,87,88,89]. However, the virus’s high mutation rate—leading to antigenic drift and occasional antigenic shift—poses a significant challenge to the immune system [86,87,88,89]. This rapid evolution of the virus limits the protective efficacy of immune responses from prior infections or vaccinations against new strains. Although memory B and T cell responses are generated, the continual evolution of the virus restricts the breadth and durability of immune protection [86,87,88,89]. Influenza vaccines must be updated annually to match circulating strains, highlighting the virus’s antigenic variability [86,87,88,89]. Current research efforts are directed towards the development of a universal influenza vaccine that targets conserved viral elements, such as the stalk domain of hemagglutinin, to induce a broader immune response that could provide cross-protection across multiple strains despite antigenic shifts [86,87,88,89].

In conclusion, RSV, SARS-CoV-2, and influenza elicit distinct immunological responses shaped by each virus’s structural and molecular adaptations. RSV’s immune evasion strategies result in incomplete immune memory, leading to frequent reinfections. SARS-CoV-2 induces a potent but sometimes pathogenic immune response, with a degree of memory that can be challenged by viral mutations. Influenza’s high mutation rate necessitates adaptive immune responses that are continuously modified, resulting in a need for annual vaccination. These variations underscore the complexities in vaccine development and immune response management for respiratory viruses, as each pathogen presents unique immunological challenges that influence both natural immunity and vaccine strategies.

### 4.5. Limitations

This study has several limitations that impact the generalizability of its findings, including variability in the study designs and sample sizes among the selected articles, which can lead to inconsistent data interpretations. Additionally, the duration of immunity assessed in the studies may not reflect long-term immunity due to the relatively recent emergence of SARS-CoV-2 and limited long-term data on RSV vaccines. Immunocompromised patients were also unevenly represented across studies, leading to potential bias in understanding the durability of immunity in these high-risk groups. The rapidly evolving viral strains, especially for SARS-CoV-2, pose challenges in assessing immune durability as new variants may alter immune response dynamics. Many studies emphasize biochemical markers like neutralizing antibody titers, which provide a measurable but incomplete picture of immunity [93,94]. While these markers are useful, they do not directly reflect clinical immunity—the ability of an immune response to protect against symptomatic disease or severe outcomes in the real-world environment [93,94]. This is visible in the divergence observed between high antibody titers post-vaccination and breakthrough infections that occur due to factors like immune evasion by viral variants [93,94]. Functional cellular immunity, involving T-cell-mediated responses, is central to durable protection, especially against respiratory viruses. However, quantifying cellular immunity remains challenging. Unlike antibody levels, T-cell responses do not have universally accepted correlates of protection and are harder to measure at scale [92,93]. Furthermore, hybrid immunity, which combines natural infection and vaccination, appears to offer robust protection through both humoral and cellular mechanisms [85]. Yet, most studies on hybrid immunity rely on observational cohorts and lack granularity in assessing its specific contributions to clinical outcomes [94].

## 5. Conclusions

In conclusion, this study underscores the complexities in achieving durable immunity through immunization against SARS-CoV-2, influenza, and RSV, particularly among immunocompromised populations. While vaccines induce robust initial immune responses, the durability of protection varies widely across different viruses and is often limited, with immunity waning more rapidly in certain vulnerable groups. Emerging evidence suggests that booster doses and hybrid immunity, combining natural and vaccine-induced immunity, may extend protection, especially as viral mutations continue to challenge vaccine efficacy. This variability in immune response highlights the importance of early diagnosis and targeted antiviral therapy in managing these infections in immunocompromised patients. The findings highlight the need for tailored vaccination strategies, including next-generation vaccines that enhance both systemic and mucosal immunity, to ensure sustained protection. Further research is essential to refine these approaches and address the specific needs of high-risk individuals, thereby enhancing public health responses to current and future respiratory pathogens.

## Figures and Tables

**Table 1:** Representative studies on the durability of adaptive immunity for immunocompetent patients across different immunity approaches.

Included Studies	Disease	Sample Size (n)	Study Design	Vaccine Type	Durability of Adaptive Immunity	Immunity Assessment
Vaccine-induced Immunity						
Ciabattini, A. 2021 (Italy) [49]	SARS-CoV-2	145	Longitudinal Cohort Study	BNT162b2 mRNA Vaccine	6 months	Memory B cell response
Zhao, W. et al. 2022 (China) [50]	SARS-CoV-2	150	Longitudinal Observational Study	CoronaVac, Inactivated COVID-19 Vaccine	<12 months	Binding and neutralizing antibody levels, cytokine production, and memory T cells
Davies, C. W. et al. 2020 (USA) [51]	Influenza	53	Longitudinal Study	Inactivated Influenza Vaccine	<12 Months	Bone marrow plasma cells, antibody response, and antibody-secreting cells
Coughlan, L. et al. 2018 (UK) [52]	Influenza	73	Phase 1 Randomized Trial	Chimpanzee Adenovirus Vector Vaccine and Modified Vaccinia Ankara Vector Vaccine	18 months	T cell response
Kampmann, B. et al. 2023 (USA) [53]	RSV	3570	Clinical Trial	RSVpreF Vaccine, Bivalent Prefusion F Protein-Based Vaccine	6 months	Time until infection
Natural Immunity						
Sherina, N. et al. 2021 (Sweden) [54]	SARS-CoV-2	88	Observational study	N/A	6–8 months	Antibody levels, memory B cells, CD8+ T cells, and CD4+ T cells
Pitiriga, V. C. et al. 2023 (Greece) [55]	SARS-CoV-2	182	Retrospective Cohort Study	N/A	>12 months	T-cell response
Sridhar, S. et al. 2014 (UK) [56]	Influenza	53	Observational study	N/A	>15 months	Hemagglutination-inhibition antibody levels
Ranjeva, S. et al. 2019 (USA) [57]	Influenza	706	Retrospective Cohort Study	N/A	50% reduction after 3.5–7 years	Hemagglutination AB levels
Blunck, B. N. et al. 2022 (USA) [58]	RSV	19	Prospective Cohort Study	N/A	3–4 months	Memory T cell response
Hybrid Immunity						
Hall, V. et al. 2022 (UK) [59]	SARS-CoV-2	35,768	Prospective Cohort Study	BNT162b2 mRNA Vaccine ChAdOx1 nCoV-19 Vaccine	51% after 6 months	Repeat PCR-confirmed infections
Goldberg, Y. et al. 2022 (Israel) [60]	SARS-CoV-2	5,724,810	Retrospective Cohort Study	BNT162b2 mRNA Vaccine	2–6 months	Repeat PCR-confirmed infections
Mazzoni, A. et al. 2021 (Italy) [61]	SARS-CoV-2	22	Observational Study	BNT162b2 mRNA Vaccine	>50 days	antibody levels and T cell activity
Bonduelle, O. et al. 2014 (France) [62]	Influenza	50	Observational study	A(H1N1)pdm09 Adjuvanted Influenza Vaccine	12 months	antibody levels and T-cell response
Lee, J. H. et al. 2019 (Korea) [63]	Influenza	124	Observational study	Quadrivalent Inactivated Subunit Influenza Vaccine	6 months	Hemagglutination antibody levels

**Table 2:** Representative studies on the durability of adaptive immunity for immunocompromised patients across different immunity approaches.

Included Studies	Disease	Sample Size (n)	Immunodeficiency	Study Design	Vaccine Type	Durability of Adaptive Immunity	Immunity Assessment
Vaccine-induced Immunity							
Szekanecz, Z. et al. 2023 (Hungary) [64]	SARS-CoV-2	6,391,634	Hematological malignancies, solid organ transplants, immunosuppressive therapy, and primary immunodeficiency	Observational Study	BNT162b2 mRNA Vaccine	3–6 months, improved with booster	Rate of confirmed infection and death
Reeg, D. B. et al. 2023 (Germany) [65]	SARS-CoV-2	279	Cancer, HIV-positive, solid organ Transplant, and immunosuppressive therapy	Observational Cohort Study	BNT162b2 mRNA Vaccine	6 months	T cell assay
Sjöwall, J. et al. 2022 (Sweden) [66]	SARS-CoV-2	12	Hematological malignancy, spondyloarthritis, solid organ transplant, and immunosuppressive therapy	Prospective Cohort Study	BNT162b2 mRNA Vaccine ChAdOx1 nCoV-19	0–6 months	Spike protein antibody levels, interferon-gamma secretion, and T-cell activation
Cho, Y. K. et al. 2023 (Korea) [67]	Influenza	60	Post-hematopoietic stem cell transplantation and post-chemotherapy	Prospective Study	Quadrivalent Inactivated Subunit Influenza Vaccine	<6 months	Hemagglutination inhibition antibody levels
Felldin, M. et al. 2014 (Sweden) [68]	Influenza	49	Solid organ transplant and immunosuppressive therapy	Prospective Cohort Study	AS03-Adjuvanted Influenza A(H1N1)pdm09 vaccineTrivalent Inactivated Subunit Influenza Vaccine (TIV/10)	<1 year	Hemagglutination inhibition antibody levels
Natural Immunity							
Kinoshita, H. et al. 2021 (USA) [69]	SARS-CoV-2	5	Primary antibody deficiency	Observational Study	N/A	3 months	Spike protein antibody levels and T-cell activation
Vigón, L. et al. 2022 (Spain) [70]	SARS-CoV-2	9	Common variable immunodeficiency, hematological malignancy, immunosuppressive therapy	Observational Cohort Study	N/A	2 months	Spike protein antibody levels, cytokine secretion, and T-cell activation
Søfteland, J. M. et al. 2021 (Sweden) [71]	SARS-CoV-2	65	Chronic immunosuppressive therapy and solid organ transplant	Longitudinal Observational Study	N/A	9 months	Spike protein antibody levels, interferon-gamma secretion, and T-cell activation
Hirzel, C. et al. 2019 (Canada) [72]	Influenza	196	Solid organ transplant	Prospective Cohort Study	N/A	4-week antibody response	Haemagglutinin inhibiting antibody response
Kim, S. R. et al. 2023 (USA) [73]	RSV	39	Hematopoietic cell transplant	Observational Study	N/A	2 months	Pre-fusion F antibody titers
Hybrid Immunity							
Nazaruk, P. et al. 2023 (Poland) [74]	SARS-CoV-2	118	Common variable immunodeficiency, X-linked agammaglobulinemia, and immunosuppressive therapy	Observational Study	BNT162b2 mRNA Vaccine	8 months	Spike protein antibody levels, interferon-gamma secretion, and T-cell activation
Rabenstein, M. et al. 2023 (Sweden) [75]	SARS-CoV-2	98	Multiple sclerosis, neuromyelitis optica spectrum disorder, and immunosuppressive therapy	Cohort study	BNT162b2 mRNA Vaccine mRNA-1273	<6 months	Spike protein antibody levels, interferon-gamma secretion, and T-cell activation
Ekström, N., et al. 2023 (Finland) [76]	SARS-CoV-2	488	Common variable immunodeficiency, solid organ transplant, hematological malignancy, and immunosuppressive therapy	Observational Cohort Study	BNT162b2 mRNA Vaccine mRNA-1273	<6 months	Spike protein antibody levels, interferon-gamma secretion, and T-cell activation
Al-Dury, S. et al. 2023 (Sweden) [77]	SARS-CoV-2	98	Rheumatoid arthritis, systemic lupus erythematosus, Psoriatic arthritis, and immunosuppressive therapy	Observational Study	BNT162b2 mRNA Vaccine mRNA-1273	>6 months	Spike protein antibody levels, interferon-gamma secretion, and T-cell activation
Mehta, L. et al. 2017 (USA) [78]	Influenza	90	Relapsing-remitting multiple sclerosis	Prospective Cohort Study	Trivalent Iinactivated Subunit Influenza Vaccine	>28 days	Seroprotection and seroconversion rates

## Data Availability

No new data were created or analyzed in this study. Data sharing is not applicable to this article.
